# Dynamic behavior and constitutive model of marble subjected to wet-dry cycling

**DOI:** 10.1371/journal.pone.0337703

**Published:** 2025-12-02

**Authors:** Yongsheng Liu, Maolin Zhai, Zheng Yang, Zhongyi He

**Affiliations:** 1 State Key Laboratory of Digital Intelligent Technology for Unmanned Coal Mining, Anhui University of Science and Technology, Huainan, China; 2 School of civil engineering and architecture, East China Jiaotong University, Nanchang, China; 3 Jiangxi Vocational college of Mechanical & Electrical Technology, Nanchang, China; Henan Polytechnic University, CHINA

## Abstract

The cyclic deterioration induced by periodic water-level fluctuations, combining dry-wet cycles and chemical corrosion, poses significant threats to the stability and durability of rock masses in reservoir areas. These effects become particularly critical under dynamic loading conditions. To investigate the mechanical characteristics and damage behavior of rock subjected to the coupled effects of dry-wet cycles, chemical corrosion, and dynamic loading, dynamic impact tests were conducted on marble specimens using a split Hopkinson pressure bar (SHPB) system. The tests considered various pH environments and different numbers of dry-wet cycles. By analyzing physical and mechanical parameters such as strength, elastic modulus, mass loss rate, and water absorption rate, and incorporating damage mechanics and the Lemaitre strain equivalence hypothesis, a dynamic constitutive model for marble was developed. The results indicate that as the number of dry-wet cycles increases, the mass loss rate, water absorption rate, peak strength, and elastic modulus undergo significant changes in the initial stages, which gradually stabilize in later stages. The degree of mechanical degradation under different chemical environments follows the order: pH = 4 > pH = 10 > pH = 7. Both dynamic compressive strength and elastic modulus increase with rising impact air pressure, demonstrating higher sensitivity to impact pressure than to pH variations or the number of dry-wet cycles. The established dynamic damage constitutive model effectively captures the stress–strain behavior of marble under dry-wet cycles and dynamic loading. The findings provide a theoretical basis for assessing the safety and stability of reservoir bank rock masses.

## Introduction

Most rock masses in nature are in complex environments. Take the rock masses in reservoir areas as an example. Phenomena such as rainfall and evaporation often occur in the environment where they are located. Additionally, affected by the change of groundwater level, the rocks undergo frequent dry-wet cycles [1,2]. Acidic or alkaline ions in the aqueous solution gradually damage the cementation structure between mineral particles through chemical reactions with rock minerals, resulting in a significant weakening of the original mineral connection inside the rock mass. Coupled with the scouring of water flow, they jointly exacerbate the damage and deterioration of the rock [3–5]. Moreover, rock mass engineering may also be subjected to dynamic disturbances—such as those induced by drilling and blasting activities—during both the construction and operational phases. Therefore, an in-depth investigation into the dynamic mechanical behavior of rocks under the coupled influence of chemical corrosion and dry-wet cycling is of paramount importance for accurately predicting and assessing the long-term stability of engineering structures.

In existing studies, numerous scholars have conducted extensive experimental investigations on rocks subjected to dry-wet cycles. These studies have demonstrated varying degrees of influence on the physical and mechanical properties of different rock types—including density, P-wave velocity [6], porosity [7], and microstructure [8]—following cyclic drying and saturation. Tao et al. [9] observed that chemical corrosion weakens the bonding between mineral grains, leading to a notable reduction in the mechanical performance of marble. Using nuclear magnetic resonance (NMR) technology, Zhang et al. [10] revealed the deterioration mechanism of marble under acidic dry-wet cycling. Liu et al. [11] investigated the mechanical behavior and energy evolution during the failure process of marble under coupled chemical corrosion and dry-wet cycles based on uniaxial compression tests. Wen et al. [12] studied the mechanical properties and failure mechanisms of red sandstone under dry-wet cycles via triaxial compression tests, analyzing rock failure mechanisms under water–rock interaction. Du et al. [13] performed systematic dynamic impact tests to examine the influence of dry-wet cycles on the mechanical behavior of red sandstone. Their results indicated that the dynamic compressive strength gradually decreases with an increasing number of dry-wet cycles, while also exhibiting strain rate dependence—that is, higher loading rates enhance the strength. Through dynamic tensile tests, Sun et al. [14] explored the effect of dry-wet cycles on the dynamic tensile properties and energy dissipation characteristics of red sandstone.

Environmental issues arising from human activities have led to the acidification or alkalization of groundwater, making the dynamic response of chemically corroded rock a critical concern in geotechnical engineering. Chemical environments with different pH levels significantly affect various rock properties, including P-wave velocity [15], mass [16], elastic modulus [17] dynamic strength [18], and failure patterns [19]. Li et al. [20] observed that chemical erosion significantly promotes microcrack propagation in limestone. Through uniaxial compression tests on acid-treated layered limestone, Mo et al. [21] reported a notable reduction in the mechanical performance of the chemically corroded specimens. Niu et al. [22] investigated the influence of chemical corrosion on the pore structure and dynamic mechanical properties of sandstone, demonstrating that prolonged corrosion duration leads to decreased peak strength and evident chemical damage.

By developing a specific mapping between stress and strain, the rock damage constitutive model quantitatively captures the random crack expansion during rock damage evolution. Sun et al. [23] developed a uniaxial damage constitutive model for dolomite influenced by water, utilizing the Weibull distribution and Mohr-Coulomb strength criterion, and validated its applicability through numerical and laboratory tests. Chen et al. [24] formulated the damage mechanics principle and symmetric normal distribution theory for alteration under dry-wet cycles, establishing the damage constitutive model parameters for altered granite under these conditions.The triaxial compressive damage ontology model developed by Chao et al. [25] accounted for the effects of damage threshold and residual strength in freeze-thawed rocks. Xu et al. [26] analyzed the deformation behavior and mechanical properties of rocks, and established a thermal damage ontology model using the energy principle, effective stress principle, and damage theory with the damage variable as the internal state variable. Additionally, damage ontology models based on statistical principles, as proposed by several researchers, have effectively characterized the damage evolution mechanisms in rocks [27–30].

While existing studies have separately investigated the effects of chemical corrosion, dry-wet cycles, or static/dynamic loading on the mechanical characteristics of rocks, and have established statistical damage constitutive models considering multiple factors, research on the mechanical response of rocks under the coupled influence of dry-wet cycles, chemical corrosion, and dynamic loading remains insufficient. To address this gap, dynamic impact tests were conducted on marble specimens under varying chemical environments and different numbers of dry-wet cycles. This study systematically analyzes the evolution of dynamic mechanical parameters, reveals the degradation mechanisms of marble under different pH environments and cyclic saturation-drying conditions, and develops a dynamic damage constitutive model based on damage mechanics. The findings provide scientific support for dynamic risk early warning and engineering protection decisions in underground engineering, hydraulic engineering, mining engineering, and related fields.

## Materials and experimental methodology

### Specimen preparation

**Fig 1:**
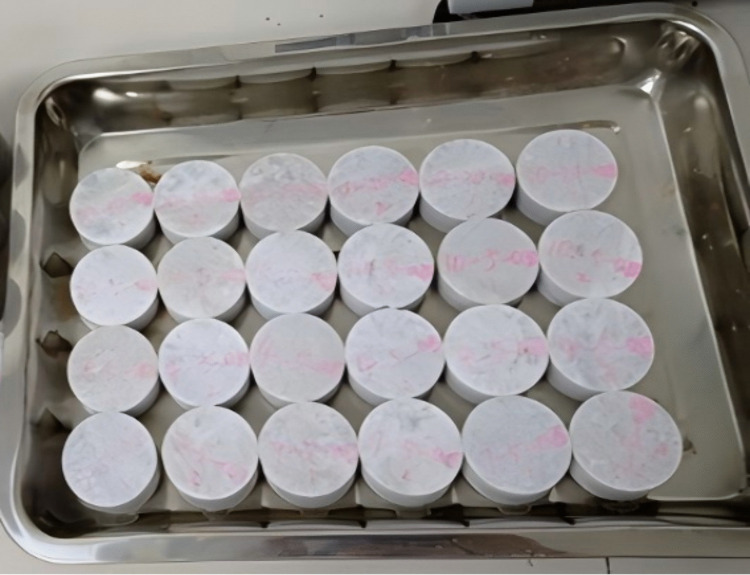
Representative prepared marble specimens.

The test specimens were obtained from Yunnan Province, China, where marble is widely distributed. To ensure the accuracy and reliability of the experimental results, all specimens were sourced from the same rock block. The marble was carefully selected from intact rock masses free of fractures and rigorously inspected to exclude surface defects. High-precision water-jet cutting was employed to process the specimens into standard cylinders with a height of 37.5 mm and a diameter of 75 mm, in accordance with the International Society for Rock Mechanics (ISRM) suggested methods for rock specimen preparation. The manufactured specimens exhibited end-face parallelism and perpendicularity deviations within ±0.02 mm. The final processed specimens are shown in Fig 1.

### Experimental setup

**Fig 2:**
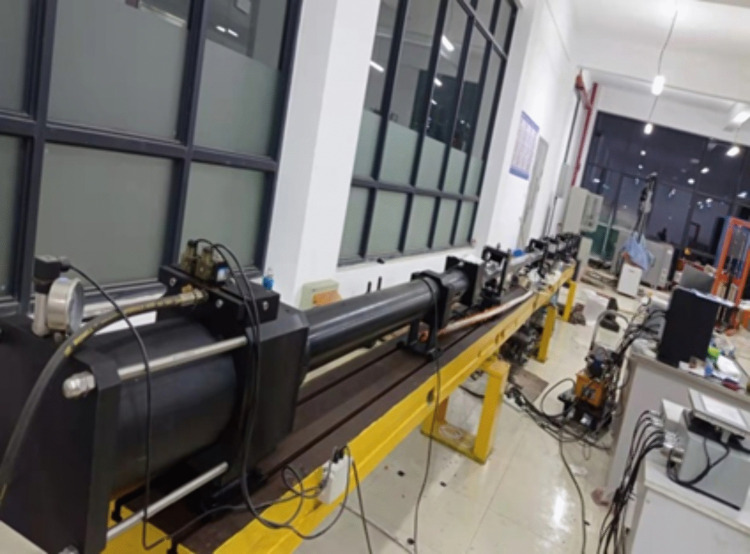
Split Hopkinson pressure bar (SHPB) experimental setup.

The SHPB experimental setup used in this study is shown in Fig 2. The system consists of three aligned bars: a striker bar (0.5 m), an incident bar (3 m), and a transmission bar (2.5 m). All bars have the same diameter of 75 mm to ensure consistent wave propagation. Prior to testing, a thin layer of vaseline was applied to both end surfaces of the specimen to minimize friction effects, and a rubber shim was attached to the impact end of the incident bar to generate smooth sinusoidal waves with reduced dispersion. Based on the one-dimensional stress wave theory and the stress uniformity assumption [31], the stress, strain, and strain rate in the specimen were calculated using the three-wave method.

### Experimental design

To investigate the potential effects of chemical environments on rock mechanical properties—particularly the degradation patterns of rock masses under acidic, neutral, and alkaline conditions—this study selected representative chemical solutions with pH values of 4, 7, and 10. To minimize data scatter, three parallel specimens were prepared for each testing condition. Prior to testing, the processed specimens were dried in an oven at 105 °C for 24 hours, cooled to room temperature, and their initial masses were recorded. The specimens were then immersed in the prepared chemical solutions for 24 hours under natural conditions. After immersion, their masses were measured again to complete one full dry–wet cycle. While the initial drying temperature was maintained at 105 °C, subsequent drying steps were conducted at 60 °C. The experiment included four stages: 0 (dry state), 5, 10, and 20 dry–wet cycles, to systematically evaluate the influence of cycle number on the mechanical degradation of marble under different chemical environments.

### Experimental procedure

Prior to formal testing, preliminary impact tests were conducted to determine appropriate impact pressures. Microcracks initiated in the specimen at an impact pressure of 0.12 MPa, while complete failure occurred at 0.18 MPa. Based on these observations, the final impact pressures for this study were selected as 0.12 MPa, 0.15 MPa, and 0.18 MPa. Given the fluctuating nature of strain rates during testing, impact pressure was adopted as the representative parameter for characterizing strain rate intensity. The corresponding relationship between these two parameters is summarized in Table 1.

**Table 1:** Correlation between impact air pressure and strain rate.

Impulse pressure (MPa)	0.12	0.15	0.18
Strain rate (s^-1^)	35.2 ~ 42.6	56.4 ~ 60.7	81.3 ~ 84.7

The standardized experimental protocol comprised: (1) system verification including Hopkinson bar integrity checks and pneumatic system calibration, followed by thorough cleaning of all bar contact surfaces; (2) A rubber pulse shaper was attached to the impact end of the incident bar to generate smooth sinusoidal waveforms with reduced dispersion. The striker bar was then propelled into the launch chamber until a stable position was achieved; (3) application of vaseline lubricant to bar-specimen interfaces, precise specimen positioning using alignment fixtures to ensure coaxial loading; (4) data acquisition system initialization at 1 MHz sampling frequency, followed by controlled gas release at predetermined pressures to generate stress waves while synchronously recording incident, reflected, and transmitted wave signals; (5) post-test specimen recovery for failure mode documentation, followed by system resetting for subsequent tests. This rigorous procedure ensured experimental repeatability across all specimen groups.

## Results analysis

### Physical characteristics of marble

To quantitatively analyze the mass loss of specimens after dry-wet cycles in chemical solutions, the mass loss rate was defined as the percentage ratio of the mass difference (before and after cycles) to the initial dry mass of the specimen. The calculation formula is given by Equation (1):


$$m_{l}=\frac{m_{\text{0}}-m_{n}}{m_{\text{0}}}\times \text{100}\%$$ (1)

Where $m_{\text{0}}$ represents the initial dry mass and $m_{n}$ denotes the mass after n wet-dry cycles.

**Fig 3:**
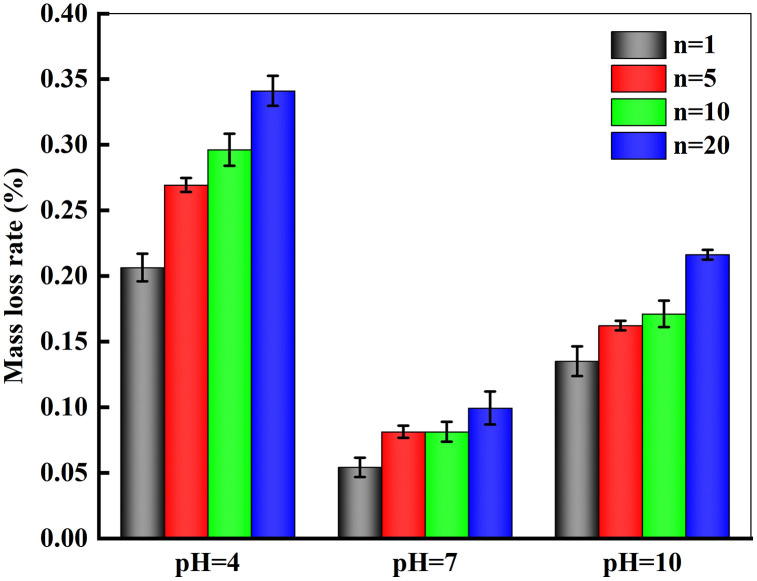
Mass Loss Rate of Specimens.

Experimental results in Fig 3 demonstrate consistent mass reduction in marble specimens across all pH conditions. Acidic solution (pH = 4) exhibited the most pronounced erosive effect, with mass loss rates persistently higher than those in neutral (pH = 7) and alkaline (pH = 10) environments throughout testing. Notably, significant mass reduction occurred during both initial and final cycles. The maximum single-cycle loss (0.198%) was observed in pH = 4 solution during the first cycle, with subsequent cycles showing gradually decreasing loss rates, culminating in a cumulative 0.341% loss after final cycling. Alkaline conditions (pH = 10) induced relatively moderate deterioration, yielding 1.2g absolute mass loss (≈0.216%) after equivalent cycles. Neutral environment (pH = 7) specimens displayed the most stable mass evolution, maintaining consistently lower loss rates with gentle linear progression.This phenomenon can be attributed to the abundance of H⁺ ions in the acidic solution, which react chemically with calcite (CaCO₃) in the marble. During testing, visible bubbles were observed on the specimen surface, and the dissolution of reaction products into the solution led to a macroscopic reduction in specimen mass. In the pH = 10 solution, minor reactions with trace SiO₂ present in the specimen also contributed to mass reduction. In contrast, under neutral conditions (pH = 7), physical processes dominated, with surface material being progressively eroded and detached during successive dry-wet cycles.

The natural water absorption of marble specimens was determined using Equation (2):


$$\omega_{a}=\frac{m_{a}-m_{d}}{m_{d}}\times \text{100}\%$$ (2)

where $m_{d}$ represents the oven-dried specimen mass and $m_{a}$ denotes the mass after natural water absorption.

**Fig 4:**
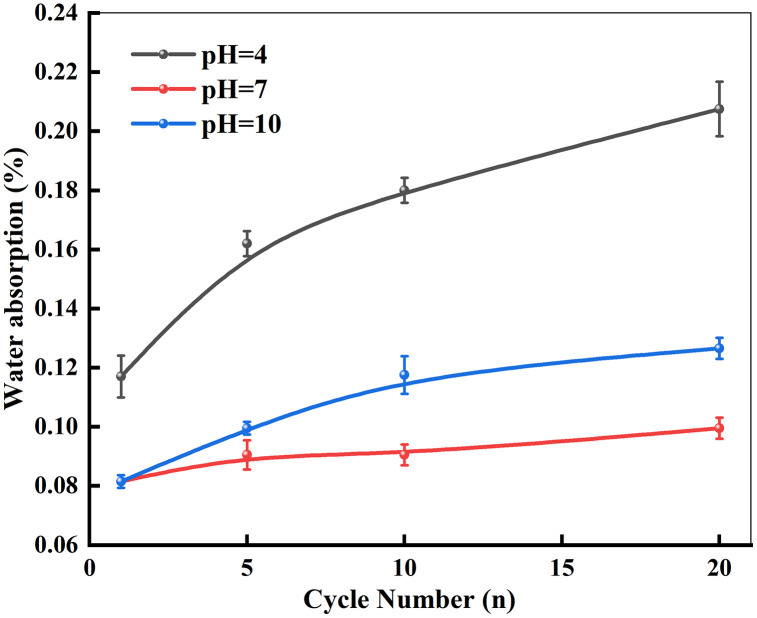
Relationship between water absorption and dry-wet cycles.


Analysis of the experimental curves in Fig 4 indicates that the relationship between the natural water absorption of marble specimens and the number of dry-wet cycles follows a typical logarithmic growth pattern. The water absorption increased markedly during the first five cycles, accounting for the dominant portion of the total increase. Beyond five cycles, the growth rate decreased and the curve gradually plateaued. The magnitude of water absorption increase varied significantly among chemical environments. Specimens immersed in the pH = 4 solution underwent pronounced chemical reactions, which facilitated the expansion of both surface and internal pores. In contrast, the limited reactive components in the pH = 10 solution resulted in slower pore development and correspondingly lower water absorption. The relationship between water absorption and the number of dry-wet cycles can be expressed by Equation (3).

$$\omega =\left\{ \;\;\;\begin{array}{c} \text{0}.\text{096}+\text{0}.\text{036ln}(\text{n}+\text{0}.\text{78})\;(\text{pH}=\text{4})\\ \text{0}.\text{085}+\text{0}.\text{003ln}(\text{n}-\text{0}.\text{58})\;(\text{pH}=\text{7})\\ \text{0}.\text{061}+\text{0}.\text{022ln}(\text{n}+\text{1}.\text{54})\;(\text{pH}=\text{10}) \end{array}\right.\;$$ (3)

### Dynamic mechanical properties of marble

#### Effect of wet-dry cycles on mechanical characteristics.

**Fig 5:**
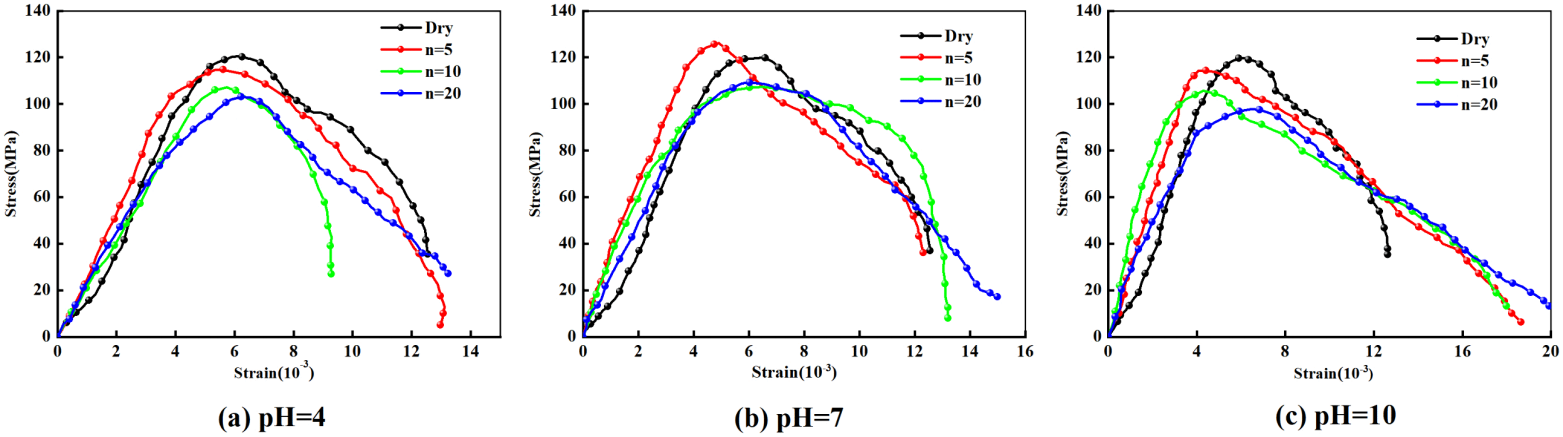
Dynamic stress-strain behavior of marble under varying wet-dry cycles.

Fig 5 demonstrates that under 0.12 MPa impact pressure, the dynamic stress-strain curves of marble specimens exhibit similar patterns across different pH values and varying numbers of wet-dry cycles. The dynamic failure process can be systematically categorized into three distinct phases: (1) elastic deformation stage, characterized by an essentially linear correlation between stress and strain, where the duration of this phase varies with the number of wet-dry cycles under identical pH conditions; (2) yielding stage, marked by nonlinear stress-strain behavior with progressive curvature toward the strain axis and decelerating stress development until reaching peak stress; and (3) failure stage, where stress undergoes abrupt degradation from the peak point.

The peak stress of the dynamic stress-strain curve for marble was defined as the dynamic peak strength, while the dynamic elastic modulus was calculated from the slope of the curve within the 40%–60% peak stress interval [27]. Using the dynamic mechanical parameters obtained under an impact pressure of 0.15 MPa, the variations in peak strength and dynamic elastic modulus of marble with the number of dry-wet cycles in different chemical environments are plotted.

**Fig 6:**
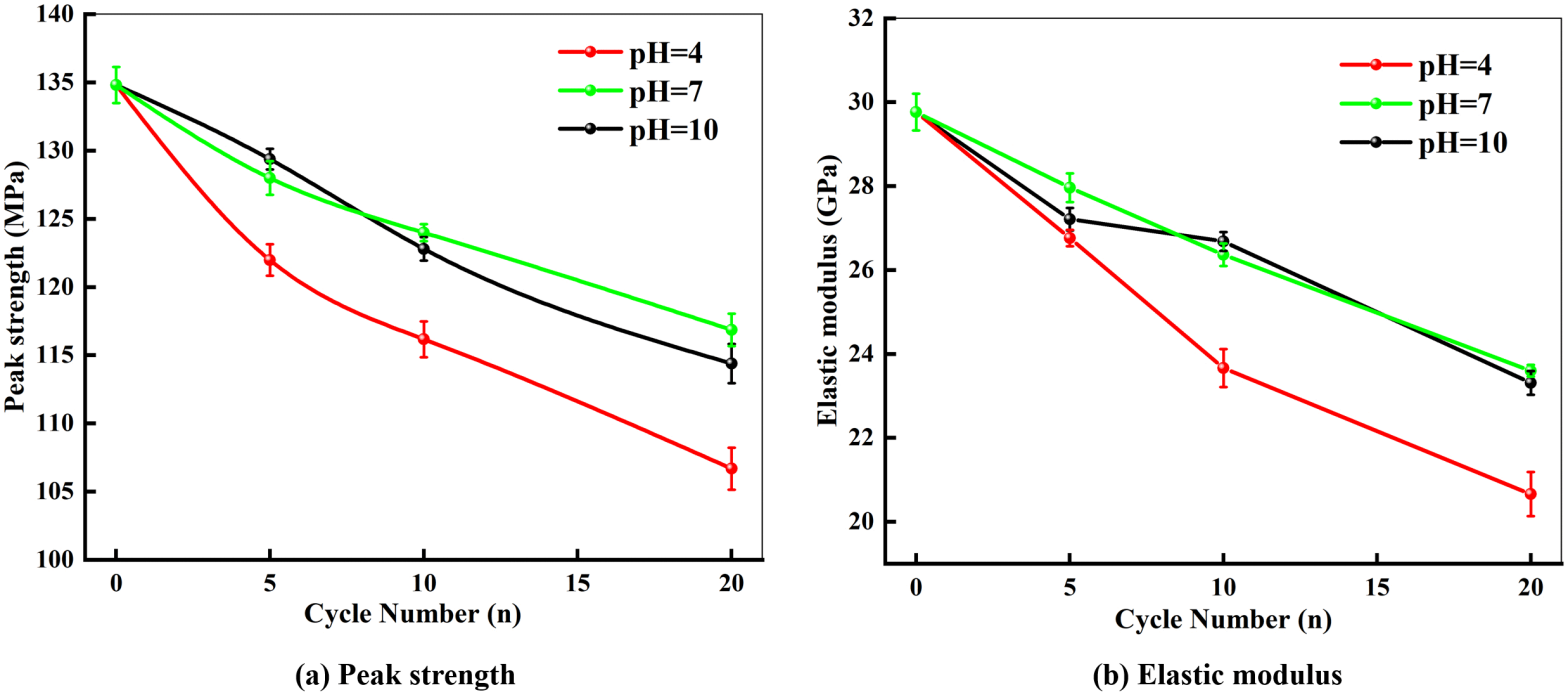
Evolution of peak strength and elastic modulus in marble under 0.15 MPa impact pressure with varying wet-dry cycles.

As shown in Fig 6, both the peak strength and elastic modulus of the specimens decreased with an increasing number of dry-wet cycles, with the most pronounced reduction occurring during the initial cycles, indicating the highest degree of damage accumulation in this stage. Taking the pH = 4 chemical environment as an example, after 5, 10, and 20 dry-wet cycles, the peak strength decreased by 9.52%, 13.83%, and 20.87%, respectively, while the elastic modulus decreased by 9.47%, 19.96%, and 30.11%, respectively. The progressively decreasing slope of the curves suggests a gradual attenuation in the rate of degradation.

Furthermore, the extent of deterioration in the dynamic mechanical parameters of marble varied under different pH environments. After 20 dry-wet cycles, specimens in pH = 4, 7, and 10 solutions exhibited peak strength reductions of 20.87%, 13.32%, and 15.15%, respectively, and dynamic elastic modulus reductions of 30.11%, 20.09%, and 21.08%, respectively. This indicates that under acidic and alkaline conditions, pre-existing pores and microcracks within the rock are further extended. The enhanced connectivity of these pathways facilitates deeper penetration of chemical solutions, increasing the likelihood of reactions with internal minerals. The cyclical interplay of physical erosion and chemical corrosion ultimately leads to an aggravated deterioration of mechanical performance.

### Influence of impact pressure on mechanical properties

**Fig 7:**
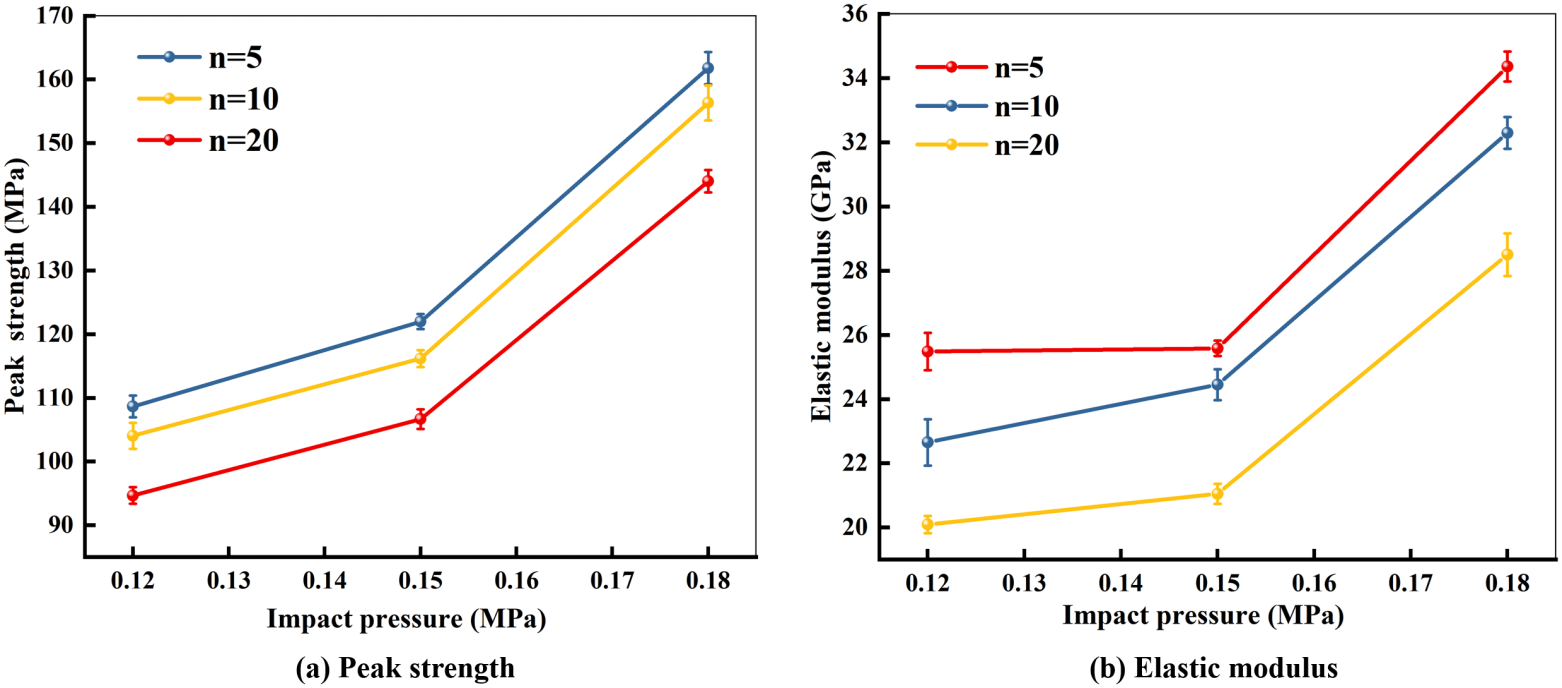
Peak strength and elastic modulus of marble under different impact pressures in pH = 4 environment.

Fig 7(a) shows the relationship between the peak strength of marble and the impact pressure after dry-wet cycles in a pH = 4 environment. Taking the specimens subjected to 5 dry-wet cycles as an example, the corresponding peak strengths under impact pressures of 0.12 MPa, 0.15 MPa, and 0.18 MPa were 108.65 MPa, 121.99 MPa, and 161.78 MPa, respectively. When the impact pressure increased from 0.12 MPa to 0.15 MPa, the strength rose by 13.34 MPa, representing an increase of 12.28%. A further increase in pressure from 0.15 MPa to 0.18 MPa resulted in a sharp strength increment of 39.79 MPa, equivalent to a 32.62% enhancement. The comparison between these two increments clearly demonstrates that higher impact pressures lead to greater peak strengths.

Fig 7(b) illustrates the variation of the dynamic elastic modulus with impact pressure in a pH = 4 environment. As shown, the dynamic elastic modulus of marble under various pH conditions generally increases with rising impact pressure but decreases with an increasing number of dry-wet cycles. Taking the specimens subjected to 10 dry-wet cycles as an example, the elastic moduli corresponding to impact pressures of 0.12 MPa, 0.15 MPa, and 0.18 MPa were 22.65 GPa, 24.45 GPa, and 32.29 GPa, respectively. Analysis of the experimental data indicates that the variation trend of the elastic modulus is generally consistent with that of the peak strength. Moreover, the rate of increase in elastic modulus becomes more pronounced at higher impact pressure levels.

**Fig 8:**
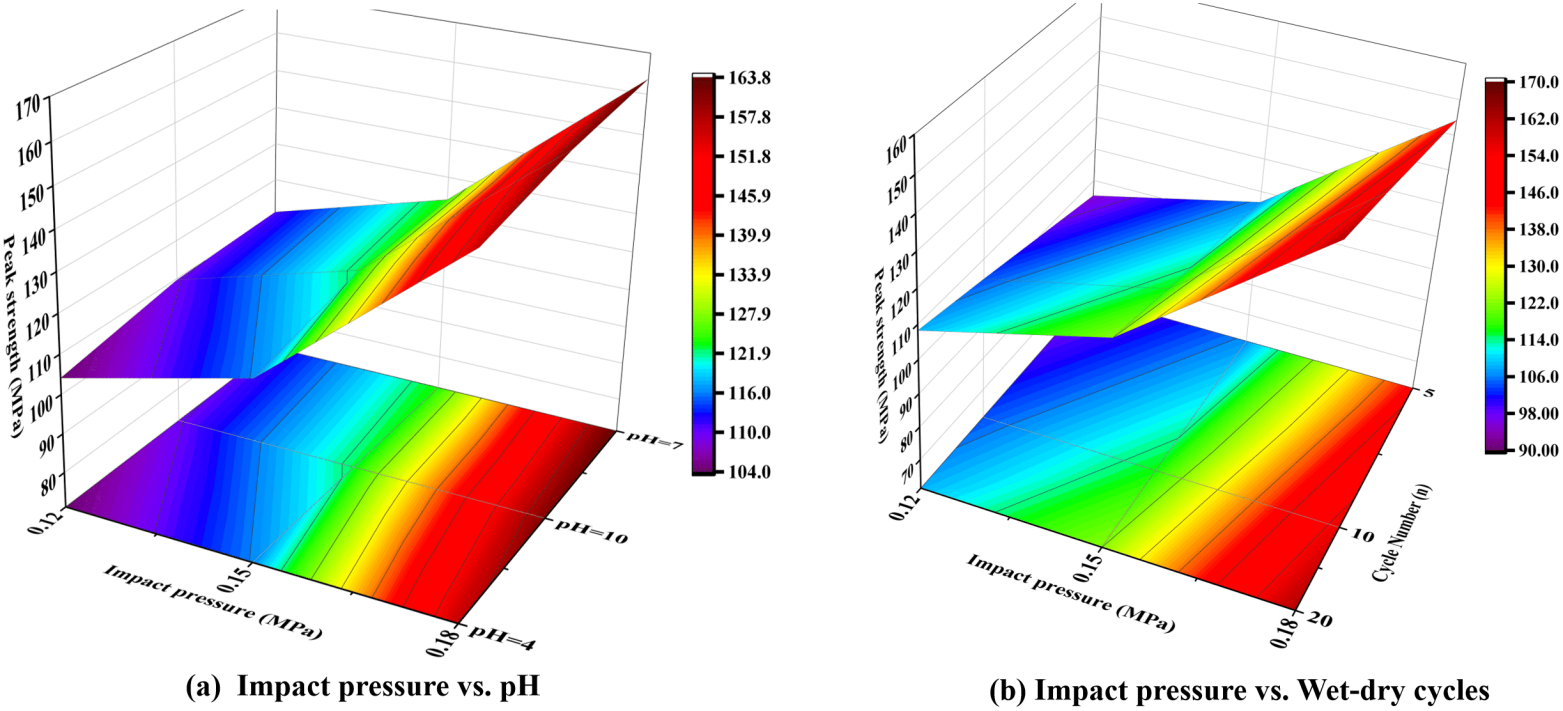
Three-dimensional surface of peak strength versus impact pressure, number of dry-wet cycles, and pH value.

The three-dimensional plot (Fig 8) provides a comprehensive comparison of the relationship between the peak strength of marble and impact pressure under different pH environments and varying numbers of dry-wet cycles. It can be visually observed that when the impact pressure increases from 0.15 MPa to 0.18 MPa, the peak strength of the specimens rises significantly. This trend indicates that the degradation effects induced by chemical corrosion and dry-wet cycles diminish as the impact pressure increases.

### Dynamic constitutive model for marble under combined wet-dry cycling and dynamic loading

#### Model formulation.

The dynamic damage of marble, resulting from the combined effects of dry-wet cycles, chemical corrosion, and dynamic loading, manifests as varying degrees of mechanical property degradation. Assuming that initial microcracks and defects are randomly distributed within the rock material, the resulting damage also follows a random distribution, leading to stochastic variations in mechanical behavior. In this study, the elastic modulus is adopted to define the damage variable of marble under dry-wet cycles, expressed as Equation (4):


$$D_{\text{1}}=\text{1}-\frac{E_{n}}{E_{\text{0}}}$$ (4)

In the formula, $D_{\text{1}}$ represents the damage variable of the rock under the action of dry-wet cycles, $E_{n}$ represents the calculated value of the elastic modulus of marble under n dry-wet cycles (n = 5, 10, 20), and $E_{\text{0}}$ is the elastic modulus of the marble specimen without experiencing dry-wet cycles.

The calculated rock damage variable $D_{\text{1}}$ is summarized in Table 2. As shown, the value of $D_{\text{1}}$ progressively increases with the number of dry-wet cycles, indicating a cumulative deterioration in the specimen’s integrity. This trend aligns consistently with the experimental observations.

**Table 2:** The variation of D_1 with the number of dry-wet cycles.

pH	Number of wet and dry cycles (n)	$D_{\text{1}}$
–	–	0
4	5	0.1446
4	10	0.2025
4	20	0.3152
7	5	0.0713
7	10	0.1245
7	20	0.2020
10	5	0.0537
10	10	0.1384
10	20	0.2340

The rock is conceptually divided into micro-units containing numerous defects. It is hypothesized that a relationship exists between the statistical distribution density of micro-unit failure and the damage variable. Extensive studies have demonstrated that the Weibull distribution effectively characterizes the evolution of damage in rock under loading. The corresponding damage variable $D_{\text{2}}$ can be expressed as:


$$D_{\text{2}}=\text{1}-exp\left[-{\left(\frac{F}{F_{\text{0}}}\right)}^{m}\right]$$ (5)

In the formula, both m and $F_{\text{0}}$ are parameters of the Weibull distribution function, and F is the external load.

At this time, the constitutive equation of the rock under the load is:


$$\text{t}\sigma =E\epsilon \left(\text{1}-D_{\text{2}}\right)=E\epsilon exp\left[-{\left(\frac{F}{F_{\text{0}}}\right)}^{m}\right]$$ (6)

The D-P criterion is selected as the judgment basis for the failure of rock micro-elements, that is:


$$F=f(\text{t}\sigma^{\prime })=\alpha_{\text{0}}I_{1}^{\prime }+\sqrt{J_{2}^{\prime }}$$ (7)

In the formula: $\alpha_{\text{0}}$ is the micro-element strength parameter, $I_{\text{1}}^{\prime }$ is the first invariant of stress, and $J_{\text{2}}^{\prime }\;$is the second invariant of the stress deviator.

Finally, all the parameters in Equation (5) can be derived as follows:


$$F=f(\text{t}\sigma {}^{\prime })=(\alpha_{\text{0}}+\frac{\text{1}}{\sqrt{\text{3}}})E\epsilon$$ (8)

$$F_{\text{0}}=(\alpha_{\text{0}}+\frac{\text{1}}{\sqrt{\text{3}}})E\epsilon_{f}m^{\frac{\text{1}}{m}}$$ (9)

$$m=-\frac{\text{1}}{\text{ln}\frac{{\text{t}\sigma }_{f}}{E\epsilon_{f}}}$$ (10)

At this time, the value of can be determined through the experimental stress-strain curve. In the formula, $\alpha_{f}$ represents the dynamic peak stress of marble without wetting-drying cycles, and $\varepsilon_{f}$ is the strain value corresponding to $\alpha_{f}$ here.

The solutions of the value of m are detailed in Table 3.

**Table 3:** The value of m under the action of each impact pressure.

impact pressure (MPa)	Dynamic peak stress (MPa)	Dynamic Peak strain ($\epsilon_{f}$)	$E_{\text{0}}$ (GPa)	m
0.12	120.09	0.0059	33.35	2.026
0.15	134.81	0.0063	36.29	1.872
0.18	175.02	0.0066	54.21	1.398

By combining Equations (5), (8), (9), and (10), the rock damage variable $D_{\text{2}}$ under the load can be solved.


$$D_{\text{2}}=\text{1}-exp\left[-{\frac{\text{1}}{m}\left(\frac{\epsilon }{\epsilon_{f}}\right)}^{m}\right]$$ (11)

According to Lemaitre’s strain equivalence principle, the strain under coupled damage conditions is equal to the sum of the strain induced by dry-wet cycling damage and the strain caused by loading damage, minus the strain in the undamaged state. That is:


$$\epsilon_{\text{12}}=\epsilon_{\text{1}}+\epsilon_{\text{2}}-\epsilon_{\text{0}}$$ (12)

In the formula: $\varepsilon_{\text{1}\text{2}}$ represents the strain following coupled damage; $\varepsilon_{\text{1}}$ denotes the strain resulting solely from dry-wet cyclic damage; $\varepsilon_{\text{2}}$ indicates the strain produced exclusively by load-induced damage, and $\varepsilon_{\text{0}}$ refers to the strain occurring without any initial damage.

Finally, the composite damage variable $D_{\text{1}\text{2}}$ can be expressed as Equation (13):


$$D_{\text{12}}=\text{1}-\frac{(\text{1}-D_{\text{1}})(\text{1}-D_{\text{2}})}{(\text{1}-D_{\text{1}}D_{\text{2}})}$$ (13)

In summary, the dynamic constitutive equation of marble under the combined action of dry-wet cycles and impact loads can be obtained as follows:


$${\text{t}\sigma =E_{\text{0}}\epsilon (\text{1}-D}_{\text{12}})=E_{\text{0}}\epsilon \frac{(\text{1}-D_{\text{1}})(\text{1}-D_{\text{2}})}{(\text{1}-D_{\text{1}}D_{\text{2}})}$$ (14)

Among them, the dynamic effect is reflected by the parameter $D_{\text{2}}$.

### Model Validation

**Fig 9:**
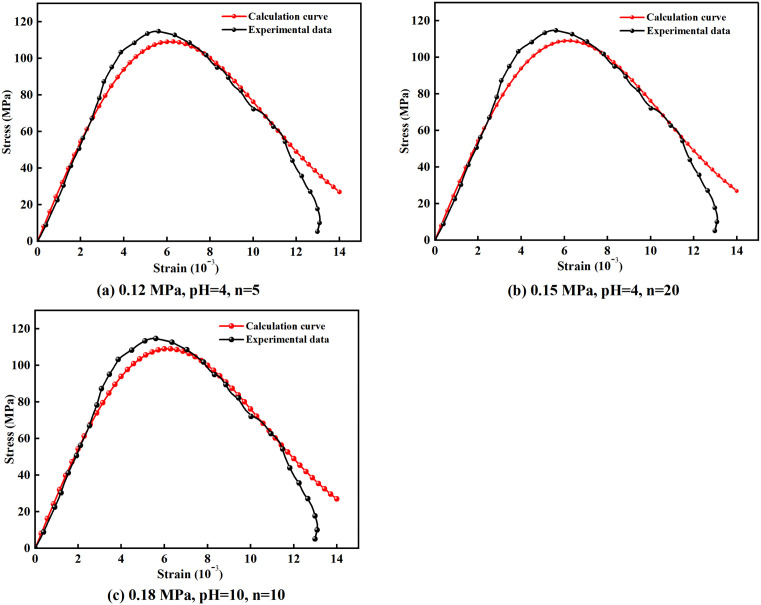
Comparison between experimental measurements and constitutive model predictions for representative specimens.

Fig 9 presents the comparative results between experimentally obtained stress-strain curves and theoretical predictions from the dynamic damage constitutive model for marble specimens subjected to varying numbers of wet-dry cycles.

As shown in Fig 9, the theoretically derived stress-strain curves exhibit good agreement with the experimental results, particularly in the pre-peak elastic stage. This consistency arises because the specimen behaves as a continuous medium before failure, enabling uniform stress distribution. However, after impact-induced failure, the rock fragments into discrete blocks, and the load-bearing mechanism shifts to the interaction of multiple fragments. The highly uncertain spatial distribution of these fragments leads to deviations between the theoretical and experimental curves in the post-peak stage.

In practical geotechnical engineering applications, monitoring and early warning typically focus on the pre-failure behavior of rock masses. The dynamic constitutive model for marble developed in this study adequately captures the mechanical response in this critical stage, thus fulfilling the essential requirements for engineering practice.

## Conclusions

This study systematically investigated the physico-mechanical properties of marble under the coupled effects of chemical corrosion, dry-wet cycles, and dynamic loading, with particular focus on the influences of chemical environment (pH), number of dry-wet cycles, and impact pressure. The main conclusions are as follows:

(1) With an increasing number of dry-wet cycles, both the mass loss rate and water absorption of marble show an increasing trend, with the most significant changes occurring during the initial cycles. Under different chemical environments, the order of mass loss rate and water absorption is: pH = 4 > pH = 10 > pH = 7.
(2) Dynamic loading tests reveal that the coupled action of chemical corrosion and dry-wet cycles leads to varying degrees of degradation in the dynamic peak strength and elastic modulus of marble. The most severe mechanical deterioration occurs in acidic environments (pH = 4), followed by alkaline conditions (pH = 10), while neutral environments (pH = 7) exhibit the least impact.
(3) Both the dynamic peak strength and elastic modulus of marble increase with rising impact pressure. Comparative analysis of the relationship between peak strength and impact pressure across different pH environments and dry-wet cycles indicates that the degradation effects induced by chemical corrosion and dry-wet cycles gradually diminish as the impact pressure increases.
(4) Based on damage mechanics theory, a coupled damage variable accounting for the combined effect of dry-wet cycles and impact loading was derived, and a corresponding dynamic damage constitutive model was established. The model effectively captures the stress-strain response of marble under simultaneous dry-wet cycles and dynamic loading, providing a theoretical reference for safety and stability assessments in environmental engineering, underground geotechnical engineering, and hydraulic engineering during both construction and operational phases.
